# Patterns and predictors of cognitive function and post-traumatic growth in cancer patients: a latent profile analysis

**DOI:** 10.3389/fpsyt.2026.1749502

**Published:** 2026-05-05

**Authors:** Libing Zhang, Riyu Huang, Meijuan Cao, Juan Fang, Dongyan Lu

**Affiliations:** School of Medicine, Huzhou University, Huzhou, Zhejiang, China

**Keywords:** cancer, cancer-related cognitive impairment, latent profile analysis, personality traits, post-traumatic growth

## Abstract

**Background:**

Cancer patients often face concurrent challenges of cancer-related cognitive impairment (CRCI) and opportunities for post-traumatic growth (PTG), but existing research primarily adopts a variable-centered approach, failing to capture the heterogeneous co-occurrence patterns of CRCI and PTG or identify key predictors of these patterns. This study aimed to explore latent profiles of psychological adaptation and their predictive factors among cancer patients, thereby responding to the practical need for refined understanding of heterogeneous adaptation patterns and providing a reference for stratified clinical interventions.

**Methods:**

A cross-sectional study was conducted between August and December 2024, recruiting 249 cancer patients from two tertiary hospitals in Zhejiang, China. Data were collected using sociodemographic and disease information, the Functional Assessment of Cancer Therapy-Cognition (FACT-Cog), the Post-Traumatic Growth Inventory (PTGI), and the Chinese Big Five Personality Inventory Brief Version (CBF-PI-B). Latent profile analysis was employed to classify patients based on standardized FACT-Cog and PTGI scores, with binary logistic regression used to identify predictors of profile membership.

**Results:**

Based on model fit indices from latent profile analysis, a two-profile solution was identified as optimal. This included the “cognitively stable but growth-constricted” profile (73.1%), characterized by better self-reported cognitive function but limited post-traumatic growth, and the “post-traumatic growth but cognitively vulnerable” profile (26.9%), featuring significant psychological growth alongside prominent perceived cognitive impairment. Binary logistic regression analysis revealed that personality traits (neuroticism, extraversion, conscientiousness, agreeableness, openness), treatment-related factors (radiotherapy, endocrine therapy), and educational level served as significant factors influencing profile membership.

**Conclusion:**

Heterogeneity exists in psychological adaptation patterns integrating CRCI and PTG among cancer patients, manifesting as two distinct profiles. Patients with lower neuroticism, higher levels of extraversion, conscientiousness, agreeableness, and openness, or those undergoing radiotherapy are more likely to belong to the “post-traumatic growth but cognitively vulnerable” profile; high educational level and endocrine therapy also show marginal positive associations with this profile. These findings support the development of tailored interventions that address these specific psychological adaptation patterns to optimize psychological care and enhance patients’ quality of life and overall well-being.

## 1 Introduction

Cancer remains a leading global public health challenge, imposing substantial socioeconomic and familial burdens worldwide. According to the 2022 global cancer statistics, an estimated 19.96 million new cancer cases were diagnosed worldwide. China bore a significant portion of this burden, accounting for 24.1% of all new cases (1). The overall upward trend in cancer incidence is largely driven by population aging, lifestyle transitions, and improved diagnostic capacity. In China, annual new diagnoses across multiple malignancies exceed 4 million, and while advancements in screening and treatment have significantly extended survival, the focus of cancer care has shifted from mere survival to optimizing patients’ quality of life and functional status during and after treatment (2). However, this extended lifespan exposes patients to a range of long-term symptomatic and psychological challenges, encompassing physiological symptoms (e.g., persistent fatigue, chronic pain, and insomnia), psychological distress (e.g., anxiety, depression, and post-traumatic stress symptoms), and cognitive impairments (e.g., cancer-related cognitive impairment)—all of which can persist for years post-treatment and undermine holistic wellbeing.

Cancer-related cognitive impairment (CRCI) is a prevalent complication across diverse cancer populations, characterized by multidimensional deficits including memory loss, attention fragmentation, slowed information processing speed, and reduced executive function. CRCI affects 30%–75% of cancer patients, with onset ranging from pre-treatment to post-treatment and persisting for years in some cases (3, 4). Among breast cancer patients, over 60% experience chemotherapy-induced cognitive impairment (CRCI) after chemotherapy (especially courses containing anthracyclines). Recent network meta-analysis shows that approximately 44% of patients report cognitive decline, while objective testing confirms cognitive impairment in approximately 21%-34%. These symptoms (such as impaired verbal memory and executive function) may persist for 3 to 5 years after treatment completion, further reducing patient adherence to subsequent adjuvant endocrine therapy (5). CRCI not only increases their disease burden but also reduces their sense of social role fulfillment, work capacity, and ability to carry out daily activities; furthermore, these cognitive impairments undermine treatment adherence and self-management capabilities—key determinants of disease prognosis—and disrupt social reintegration (e.g., returning to work), ultimately creating cascading impacts on patients’ daily lives and family systems (6–8). Despite its prevalence, CRCI is often overlooked in multi-cancer research, with most studies focusing on single malignancies or treating patients as a homogeneous group.

However, during cancer treatment, positive outcomes may also emerge, such as post-traumatic growth (PTG), which serves as a marker of resilient adaptation across cancer types. PTG encompasses five core dimensions: life perception, personal strength, new possibilities, interpersonal relationships, and self-transformation, arising through active cognitive integration of traumatic experiences, involving processes such as cognitive appraisal, ruminative thinking, and meaning-making (9, 10). This integration enables patients to reevaluate life priorities, strengthen interpersonal connections, and identify new pathways in life. PTG has been observed in a significant proportion of cancer survivors; for instance, a systematic review focusing on colorectal cancer survivors highlighted the manifestation of PTG and also observed PTG within survivor-caregiver dyads, underscoring the role of social support in fostering growth (11). Higher levels of PTG are often associated with better psychological outcomes, highlighting its clinical value for holistic cancer care (12). Theoretically, PTG relies on intact cognitive function: patients must process traumatic information, retain meaningful insights, and adjust belief systems—suggesting a potential interplay where CRCI may shape PTG trajectories by impairing these underlying cognitive processes. Yet, existing research primarily explores PTG in relation to negative psychological states rather than CRCI, leaving the interplay between CRCI and PTG unexamined in multi-cancer populations (13).

Although the individual factors influencing CRCI and PTG have been extensively studied, there remains a significant knowledge gap regarding the factors that determine their co-occurring patterns. The predominant use of a variable-centered approach in existing literature has effectively delineated linear relationships but often obscures the substantial heterogeneity in how patients experience the interplay between CRCI and PTG. This approach fails to recognize distinct combinations of CRCI and PTG (14). Moreover, research in this area has often been conducted within the confines of single cancer types. While this allows for depth, it limits the generalizability of findings and hinders the development of integrated care models that can address the dual challenges of cognitive challenges and growth opportunities across the broader cancer survivor population.

To address these limitations, Latent Profile Analysis (LPA) is a statistical technique that categorizes individuals into distinct groups based on objective adaptation indicators, thereby exploring the heterogeneity within populations with similar characteristics (15), which holds significant implications. This approach has already demonstrated utility in oncology research, such as classifying emotional inhibition subtypes in gastrointestinal cancer patients and psychological resilience profiles in elderly colorectal cancer survivors (16, 17). Therefore, LPA can be utilized as a tool to identify high-risk individuals for implementing tailored intervention strategies, assisting in the exploration of different patterns of CRCI and PTG in cancer patients. Personality traits serve as a pivotal factor in the process of adapting to cancer. Systematic reviews substantiate that stable traits, alongside adaptive coping strategies, facilitate PTG (18). While the influence of personality on cognitive function is well-established in geriatric populations, empirical evidence specifically linking it to CRCI remains scarce, highlighting a critical knowledge gap in psycho-oncology (19).

This contrast underscores the need to investigate how personality configurations shape distinct profiles of both psychological growth and cognitive function.

The Pearlin Stress Process Model provides a robust framework for conceptualizing how predisposing characteristics shape differential health outcomes in the face of illness-related stressors. This model posits that individuals’ resources and vulnerabilities, present prior to and during a stress experience, determine their patterns of adaptation (20). In the context of cancer, the diagnosis and its treatment constitute primary stressors, while outcomes manifest in diverse domains, including cognitive function (CRCI) and psychological well-being (PTG). This study positions personality traits, sociodemographic characteristics, and clinical disease characteristics as key predisposing factors hypothesized to determine an individual’s membership in distinct latent profiles of CRCI and PTG. This study thus aims to first identify these profiles and then determine how these factors predict profile membership.

## 2 Materials and methods

### 2.1 Design and sample

A cross-sectional study was conducted between August 2024 and December 2024, recruiting cancer patients from two tertiary care hospitals in Zhejiang Province, China, using a convenience sampling method. The inclusion criteria were as follows: 1) aged 18 years or older; 2) a confirmed diagnosis of malignant tumor via cytological or histopathological examination; 3) being conscious and able to communicate effectively; 4) voluntarily provided informed consent. The exclusion criteria were: 1) a history of mental illness, dementia, or severe cognitive impairment; 2) severe comorbid physical conditions; 3) a diagnosis of primary or metastatic malignant central nervous system tumors. The study protocol was approved by the Ethics Committee of Huzhou University (Approval No. 202406-03).

An *a priori* power analysis was conducted using G*Power 3.1 for regression models (α = 0.05, two-tailed), based on Cohen’s effect size conventions (21). This analysis indicated that 128 participants would be required to achieve 95% power for a medium effect size (f² = 0.15). We distributed 300 questionnaires to account for potential attrition and invalid responses. After data cleaning, 249 valid responses were retained (83.0% valid response rate). This final sample exceeds the requirement identified by the power analysis.

Furthermore, the final sample size (N = 249) is well aligned with methodological recommendations for LPA. Simulation studies suggest that samples of approximately 200 are typically sufficient for reliable profile identification when clear separation exists between profiles (22, 23). This condition was strongly met in our study, with the optimal model demonstrating excellent classification accuracy (entropy = 0.982) and strong separation (profile distance d = 1.23). Both values substantially exceed common recommendations in the LPA literature (entropy > 0.8; profile distance d > 0.7), which are considered indicative of high-quality classification (24). Therefore, the sample is validated as sufficient for both robust regression and stable LPA.

### 2.2 Instruments

#### 2.2.1 Sociodemographic and disease information

Based on an extensive literature review and discussion, the research team designed a questionnaire concerning sociodemographic and disease characteristics. The questionnaire covered the following items: age, gender, education level, marital status, occupation, place of residence, monthly household income, medical insurance, family history of cancer, cancer site, tumor stage, and comorbid chronic conditions.

#### 2.2.2 Functional assessment of cancer therapy-cognitive scale

This tool was originally developed by Wagner et al. (25) and validated in Chinese by Cheung et al. (26), was used to evaluate self-perceived cognitive function in cancer patients. It consists of 37 items across 4 domains: perceived cognitive impairments (PCI), others’ ratings (Oth), perceived cognitive abilities (PCA), and impact on quality of life (QoL), with 33 items contributing to the total score. Responses are rated on a 5-point Likert scale (0 = “never or not at all” to 4 = “several times a day or very much”). All domains except PCA are reverse-scored, with higher total scores indicating better cognitive function. In this study, the Cronbach’s α coefficient for the scale was 0.863.

#### 2.2.3 Post-traumatic growth inventory scale

This scale, originally developed by Tedeschi and Calhoun (27) and adapted into Chinese by Wang et al. (28), assesses positive psychological changes after traumatic experiences. It includes 20 items in 5 domains: life perception, personal strength, new possibilities, interpersonal relationships and self-transformation. Responses are scored on a 6-point Likert scale (0 = “ not at all” to 5 = “very much”), with higher total scores indicating greater post-traumatic growth. The Cronbach’s α coefficient in this study was 0.941.

#### 2.2.4 Chinese big five personality inventory brief version scale

This scale, developed by Wang et al. (29), measures personality traits across 5 dimensions: neuroticism, extraversion, conscientiousness, agreeableness, and openness. It comprises 40 items (7 reverse-scored), with responses on a 6-point Likert scale (1 = “completely inconsistent” to 6 = “completely consistent”). Scores are calculated for each dimension, with higher scores reflecting more prominent traits. In this study, Cronbach’s α coefficients for the dimensions ranged from 0.937 to 0.950.

### 2.3 Data collection and quality control

Data collection was performed by a research team that underwent standardized training. Prior to the survey, the researchers provided all participants with a detailed explanation of the study’s purpose, procedures, and the measures in place to ensure data confidentiality. Written informed consent was obtained from all participants before they proceeded with the questionnaires. Participants completed the questionnaires independently on-site, and the forms were collected immediately upon completion. The research team conducted an on-site verification for completeness and clarity, promptly requesting participants to supplement any missing information and clarifying any ambiguous responses through immediate follow-up questions. All questionnaires were anonymized using unique identification codes to protect participant privacy. Following data collection, a dual-entry verification process was implemented by two independent researchers. Any discrepancies identified during this process were resolved by referring back to the original questionnaires, thereby ensuring the authenticity and accuracy of the final dataset.

### 2.4 Data analysis

All statistical analyses were performed using R (version 4.5.1; R Core Team, 2025) and IBM SPSS Statistics (version 26.0), with a two-tailed significance level of α = 0.05 applied to all tests. Descriptive and univariate comparative analyses were conducted using SPSS. Continuous variables were assessed for normality using the Shapiro-Wilk test. Normally distributed variables were described using means and standard deviations (SD) and compared between groups using independent samples t-tests or one-way ANOVA, as appropriate. Non-normally distributed variables were summarized using medians and interquartile ranges (IQR) and compared using the Mann-Whitney U or Kruskal-Wallis H tests. Categorical variables were presented as frequencies and proportions (percentages) and compared using the Chi-square test or Fisher’s exact test.

LPA was performed in R to identify patient subgroups based on standardized (Z-)scores of the four FACT-Cog subscales and five PTGI domains, which were standardized to enhance interpretability and model convergence. The mclust package was used to estimate models ranging from 1 to 5 latent profiles. Model fit was evaluated using Akaike’s Information Criterion (AIC), Bayesian Information Criterion (BIC), and the sample-size-adjusted BIC (aBIC), with lower values indicating superior fit. Classification accuracy was assessed using entropy, where values closer to 1 represent more precise classification (30). The tidyLPA package was employed to conduct the Bootstrap Likelihood Ratio Test (BLRT) and the Lo-Mendell-Rubin Test (LMRT) to compare the fit of the k-profile model against the (k-1)-profile model. A significant result (p<0.05) supported the retention of the k-profile model (30). Clinical interpretability was also a key consideration in the final model selection.

To identify predictors of profile membership, binary logistic regression with backward stepwise selection (based on AIC) was performed, with the “cognitively stable but growth-constricted” profile as the reference group. Guided by the Pearlin Stress Process Model (20), the initial variable pool included all variables from three theoretically relevant domains regardless of their univariate p-values: personality traits (neuroticism, conscientiousness, agreeableness, openness, extraversion), sociodemographic factors (education level, per capita monthly household income, medical insurance), and key clinical confounders (cancer site, clinical stage, treatment modality). Time since diagnosis was included as a categorical variable (within 12 months, 13–24 months, 25–36 months, 37–48 months, over 48 months); however, time since treatment completion would have been a more precise indicator of recovery phase and cognitive impact, but these data were not available. Categorical variables were entered as factors with predefined reference levels. Multicollinearity was assessed prior to model fitting using variance inflation factors (VIF); all variables in the final model had VIF values below 5, indicating no substantial multicollinearity.

### 2.5 Ethical principles

This study was approved by the Ethics Committee of the School of Medicine at Huzhou University (Approval No. 202406-03) and was conducted in accordance with the principles of the Declaration of Helsinki. Written informed consent was obtained from all participants prior to their inclusion in the study.

## 3 Results

### 3.1 Participant characteristics

The median age of participants was 53 years (IQR 41-65). The majority were female (55.0%), married (81.5%), and had an education level of junior high school or lower (41.0%). Nearly half of the participants resided in urban areas (49.4%); 45.0% were covered by employee medical insurance, 40.6% had a family history of cancer, and 47.4% had comorbid chronic diseases. Additional sociodemographic and clinical characteristics are presented in **Table 1**.

**Table 1:** Sociodemographic and clinical disease characteristics of participants (N = 249).

Variables	M (Q1, Q3) or n (%)	Variables	M (Q1, Q3) or n (%)
Age (years)	53.0(41.0, 65.0)	Cancer site	
Gender,		Lung	5.0 (2.0%)
Male	112.0 (45.0%)	Breast	25.0 (10.0%)
Female	137.0 (55.0%)	Colorectal	20.0 (8.0%)
Education level		Thyroid	39.0 (15.7%)
Junior high school or lower	102.0 (41.0%)	Liver	27.0 (10.8%)
High school or vocational school	66.0 (26.5%)	Stomach	39.0 (15.7%)
College and above	81.0 (32.5%)	Esophageal	41.0 (16.5%)
Marital status		Cervical	25.0 (10.0%)
Married	203.0 (81.5%)	Prostate	23.0 (9.3%)
Single	20.0 (8.0%)	Other	5.0 (2.0%)
Divorced	13.0 (5.2%)	Tumor stage,	
Widowed	13.0 (5.2%)	I stage	67.0 (26.9%)
Occupational status		II stage	67.0 (26.9%)
Manual labor	96.0 (38.6%)	III stage	70.0 (28.1%)
Mental labor	66.0 (26.5%)	IV stage	37.0 (14.9%)
Unemployed	27.0 (10.8%)	Unclear	8.0 (3.2%)
Retired	50.0 (20.1%)	Comorbid chronic conditions	
Other	10.0 (4.0%)	Yes	118.0 (47.4%)
Place of residence		No	131.0 (52.6%)
Rural	126.0 (50.6%)	Time since diagnosis	
Urban	123.0 (49.4%)	Within 12 months	58.0 (23.3%)
Per capita monthly household income		13–24 months	59.0 (23.7%)
<2,000 RMB	32.0 (12.9%)	25–36 months	86.0 (34.5%)
2,000-5,000 RMB	74.0 (29.7%)	37–48 months	30.0 (12.0%)
5,001-10,000 RMB	89.0 (35.7%)	Over 48 months	16.0 (6.4%)
>10,000 RMB	54.0 (21.7%)	Treatment methods	
Medical insurance		Surgery	142(57.0%)
Self-payment	36.0 (14.5%)	Chemotherapy	138(55.4%)
Resident basic medical insurance	101.0 (40.6%)	Radiotherapy	132(53.0%)
Employee basic medical insurance	112.0 (45.0%)	Endocrine	132(53.0%)
Family history of cancer		Others	102(40.9%)
Yes	101.0 (40.6%)		
No	148.0 (59.4%)		

### 3.2 Latent profile determination

LPA was conducted based on standardized scores of the four FACT-Cog subscales and five PTGI domains in cancer patients. Starting with a single profile, models were progressively expanded to encompass 1 to 6 profiles. Through a comprehensive evaluation of model fit indices and clinical interpretability, the two-profile solution was determined to be optimal based on the following evidence (1): Among the six estimated models, the two-profile model demonstrated substantially lower BIC (5359.862) and AIC (4976.46) values compared to the one-profile model, along with excellent entropy (0.982 > 0.8), indicating high classification accuracy (2); Both the LMRT and BLRT tests yielded statistically significant results (P < 0.001). However, when the number of profiles increased to five, although this model demonstrated marginally lower BIC and AIC values, it resulted in less balanced class proportions and reduced clinical interpretability. Consequently, the two-profile model was chosen for its favorable balance of statistical fit and clinical relevance. Detailed model fit statistics are presented in **Table 2**.

**Table 2:** Fit indices for the six models using LPA (N = 249).

Model	AIC	BIC	aBIC	Entropy	LMR (P)	BLRT (P)	Profile prevalence
1	5393.44	5583.383	5614.001	–	–	–	1.000
2	4976.46	5359.862	5532.38	0.982	<0.001	<0.001	0.731/0.269
3	5119.599	5436.169	5539.84	0.871	1.0000	1.0000	0.510/0.382/0.108
4	4868.573	5259.011	5440.5	0.933	<0.001	<0.001	0.369/0.454/0.088/0.088
5	4786.551	5243.82	5532.464	0.944	<0.001	<0.001	0.398/0.289/0.153/0.072/0.088
6	4803.334	5309.847	5711.385	0.95	1.0000	<0.001	0.410/0.076/0.133/0.076/0.193/0.112

### 3.3 Characteristics and naming of latent profiles

**Figure 1:**
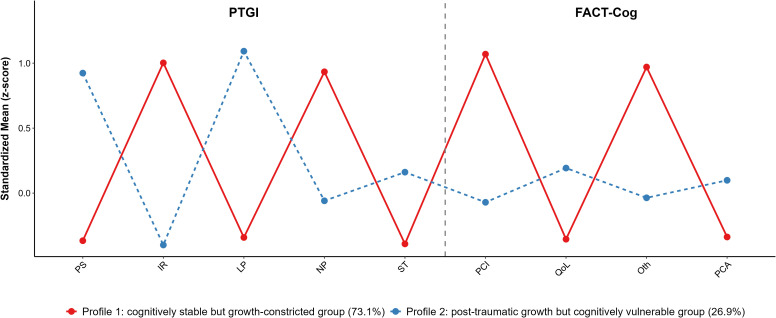
Z-scores for subscales of PTGI and FACT-Cog for the two-class latent profile model. Note: PTGI dimensions in order: Personal Strength (PS), Interpersonal Relationships (IR), Life Perception (LP), New Possibilities (NP), Self-Transformation (ST). FACT-Cog dimensions in order: Perceived Cognitive Impairments (PCI), Impact on Quality of Life (QoL), Others’ Ratings (Oth), Perceived Cognitive Abilities (PCA).

The standardized scores for the PTGI and FACT-Cog subscales in the two distinct profiles are shown in **Figure 1**. In Profile 1, patients showed higher scores across all FACT-Cog subscales, indicating better self-reported cognitive function, less impact on quality of life, and more positive ratings from others regarding their cognitive status. However, they scored lower across all PTGI domains, reflecting limited growth in areas such as personal strength, interpersonal relationships, and life meaning. This profile consisted of 182 patients, representing 73.1% of the total sample. Their overall characteristics suggest preserved cognitive function but limited post-traumatic psychological growth, hence this group was named the “cognitively stable but growth-constricted group.” In Profile 2, patients demonstrated the opposite pattern to Profile 1, with higher scores across all PTGI domains, indicating significant psychological growth following their cancer experience, including greater personal strength, deeper interpersonal connections, and more positive life perspectives. However, their FACT-Cog subscale scores were generally lower, suggesting self-reported cognitive difficulties, reduced quality of life, and negative ratings from others regarding their cognitive function. This profile comprised 67 patients, accounting for 26.9% of the total sample. Their core characteristic is significant psychological growth alongside prominent perceived cognitive impairment, leading to the name “post-traumatic growth but cognitively vulnerable group.

### 3.4 Univariate analysis of factors associated with PTGI and FACT-Cog profiles

Univariate analysis was conducted using the two latent profiles of PTGI and FACT-Cog as dependent variables, with sociodemographic characteristics, clinical features, and personality traits as independent variables. The results revealed statistically significant differences (P < 0.05) between the two profiles based on educational level (χ² = 13.849, P = 0.001), per capita monthly household income (χ² = 11.382, P = 0.010), medical insurance (χ² = 16.254, P < 0.001), time since diagnosis (Fisher’s exact test, P = 0.021), and all big five personality traits: neuroticism (Cohen’s d = 1.047, P < 0.001), conscientiousness (Cohen’s d = -1.017, P < 0.001), agreeableness (Cohen’s d = -0.856, P < 0.001), openness to experience (Cohen’s d = -1.051, P < 0.001), and extraversion (Cohen’s d = -0.942, P < 0.001) (**Table 3**). Specifically, compared to Profile 1, Profile 2 was characterized by higher education level, higher income, longer time since diagnosis, lower neuroticism, and higher conscientiousness, agreeableness, openness, and extraversion. The direction of effect sizes indicates that positive Cohen’s d for neuroticism reflects higher scores in Profile 1, whereas negative values for the other traits reflect higher scores in Profile 2.

**Table 3:** Univariate analysis of factors associated with PTGI and FACT-Cog profiles among cancer patients (N = 249).

Variables	Profile 1: cognitively stable but growth-constricted group (n = 182)	Profile 2: post-traumatic growth but cognitively vulnerable group(n = 67)	Test statistic	*P*	Effect size
Age	54.0 (43.0, 66.0)	51.0 (36.0, 60.0)	7083.000^a^	0.051	0.299
Gender,			1.571^c^	0.210	0.089
Male	77.0 (42.3%)	35.0 (52.2%)			
Female	105.0 (57.7%)	32.0 (47.8%)			
Education level			13.849^c^	0.001	0.236
Junior high school or lower	87.0 (47.8%)	15.0 (22.4%)			
High school or vocational school	45.0 (24.7%)	21.0 (31.3%)			
College and above	50.0 (27.5%)	31.0 (46.3%)			
Marital status			^b^	0.347	0.114
Married	148.0 (81.3%)	55.0 (82.1%)			
Single	13.0 (7.1%)	7.0 (10.4%)			
Divorced	9.0 (4.9%)	4.0 (6.0%)			
Widowed	12.0 (6.6%)	1.0 (1.5%)			
Occupational status			^b^	0.179	0.161
Manual labor	72.0 (39.6%)	24.0 (35.8%)			
Mental labor	41.0 (22.5%)	25.0 (37.3%)			
Unemployed	22.0 (12.1%)	5.0 (7.5%)			
Retired	40.0 (22.0%)	10.0 (14.9%)			
Other	7.0 (3.8%)	3.0 (4.5%)			
Place of residence			0.029^c^	0.865	0.020
Rural	91.0 (50.0%)	35.0 (52.2%)			
Urban	91.0 (50.0%)	32.0 (47.8%)			
Per capita monthly household income			11.382^c^	0.010	0.214
<2,000 RMB	28.0 (15.4%)	4.0 (6.0%)			
2,000-5,000 RMB	61.0 (33.5%)	13.0 (19.4%)			
5,001-10,000 RMB	58.0 (31.9%)	31.0 (46.3%)			
>10,000 RMB	35.0 (19.2%)	19.0 (28.4%)			
Medical insurance			16.254^c^	<0.001	0.255
Self-payment	35.0 (19.2%)	1.0 (1.5%)			
Resident basic medical insurance	76.0 (41.8%)	25.0 (37.3%)			
Employee basic medical insurance	71.0 (39.0%)	41.0 (61.2%)			
Family history of cancer			0.009^c^	0.925	0.015
Yes	73.0 (40.1%)	28.0 (41.8%)			
No	109.0 (59.9%)	39.0 (58.2%)			
Cancer site			^b^	0.768	0.145
Lung	3.0 (1.6%)	2.0 (3.0%)			
Breast	19.0 (10.4%)	6.0 (9.0%)			
Colorectal	15.0 (8.2%)	5.0 (7.5%)			
Thyroid	27.0 (14.8%)	12.0 (17.9%)			
Liver	22.0 (12.1%)	5.0 (7.5%)			
Stomach	27.0 (14.8%)	12.0 (17.9%)			
Esophageal	29.0 (15.9%)	12.0 (17.9%)			
Cervical	17.0 (9.3%)	8.0 (11.9%)			
Prostate	20.0 (11.0%)	3.0 (4.5%)			
Other	3.0 (1.6%)	2.0 (3.0%)			
Tumor stage,			^b^	0.080	0.185
I stage	55.0 (30.2%)	12.0 (17.9%)			
II stage	48.0 (26.4%)	19.0 (28.4%)			
III stage	51.0 (28.0%)	19.0 (28.4%)			
IV stage	25.0 (13.7%)	12.0 (17.9%)			
Unclear	3.0 (1.6%)	5.0 (7.5%)			
Treatment modality					
Surgery	101.0 (55.5%)	41.0 (61.2%)	0.437^c^	0.5084	0.051
Chemotherapy	103.0 (56.6%)	35.0 (52.2%)	0.220^c^	0.6388	0.039
Radiotherapy	99.0 (54.4%)	33.0 (49.3%)	0.334^c^	0.5634	0.046
Endocrine	48.0 (26.4%)	24.0 (35.8%)	1.692^c^	0.1934	0.092
Others	73.0 (40.1%)	29.0 (43.3%)	0.094^c^	0.7593	0.029
Comorbid chronic conditions			0.251^c^	0.617	0.041
Yes	84.0 (46.2%)	34.0 (50.7%)			
No	98.0 (53.8%)	33.0 (49.3%)			
Time since diagnosis			^b^	0.021	0.221
Within 12 months	46.0 (25.3%)	12.0 (17.9%)			
13–24 months	49.0 (26.9%)	10.0 (14.9%)			
25–36 months	59.0 (32.4%)	27.0 (40.3%)			
37–48 months	21.0 (11.5%)	9.0 (13.4%)			
Over 48 months	7.0 (3.8%)	9.0 (13.4%)			
Personality traits					
Neuroticism	21.0 (15.0, 33.0)	15.0 (13.0, 16.0)	9326.000^a^	<0.001	1.047
Conscientiousness	29.0 (16.0, 35.0)	35.0 (34.0, 37.0)	2974.000^a^	<0.001	-1.017
Agreeableness	35.0 (27.0, 38.0)	38.0 (36.0, 39.0)	3146.000^a^	<0.001	-0.856
Openness	28.0 (16.0, 33.0)	34.0 (32.0, 36.0)	2855.000^a^	<0.001	-1.051
Extraversion	28.0 (21.0, 33.0)	34.0 (31.0, 36.0)	3163.500^a^	<0.001	-0.942

^a^ Wilcoxon rank-sum test, ^b^ Fisher’s exact test, ^c^ chi-square test. Effect sizes: For continuous variables, Cohen’s d is reported (positive values indicate higher scores in Profile 1 than Profile 2, negative values indicate lower scores in Profile 1). For categorical variables, Cramer’s V is reported; the direction of association can be inferred by comparing the percentage distributions between the two profiles.

### 3.5 Binary logistic regression analysis of predictors for PTGI and FACT-Cog profiles

The backward selection procedure identified an optimal model with 8 predictors. Among the Big Five personality traits, extraversion (OR = 1.564, 95% CI: 1.295–1.888, P < 0.001), openness to experience (OR = 1.684, 95% CI: 1.289–2.209, P = 0.0001), conscientiousness (OR = 1.540, 95% CI: 1.236–1.917, P = 0.0001), and agreeableness (OR = 1.346, 95% CI: 1.126–1.610, P = 0.0011) were significant positive predictors, whereas neuroticism (OR = 0.674, 95% CI: 0.570–0.796, P < 0.001) was a significant negative predictor. Regarding treatment factors, radiotherapy (OR = 5.264, 95% CI: 1.033–26.850, P = 0.0458) was a significant positive predictor, while endocrine therapy (OR = 5.308, 95% CI: 0.993–28.320, P = 0.0510) and high education level (college and above vs. junior high or lower: OR = 5.963, 95% CI: 0.985–36.210, P = 0.0521) showed marginal positive trends (**Table 4**).

**Table 4:** Binary logistic regression analysis of predictors for PTGI and FACT-Cog profiles among cancer patients (N = 249).

Variables	Profile 1 vs Profile 2			
	β	*P*	OR	95% CI
Personality traits				
Neuroticism	-0.395	<0.001	0.674	0.570 - 0.796
Conscientiousness	0.432	0.0001	1.540	1.236 - 1.917
Agreeableness	0.297	0.0011	1.346	1.126 - 1.610
Openness	0.521	0.0001	1.684	1.289 - 2.209
Extraversion	0.447	<0.001	1.564	1.295 - 1.888
Education level (Reference: Junior high school or lower)				
High school or vocational school	0.738	0.4007	2.092	0.374 - 11.670
College and above	1.786	0.0521	5.963	0.985 - 36.210
Treatment modality (Reference: without corresponding treatment)				
Radiotherapy	1.661	0.0458	5.264	1.033 - 26.850
Endocrine therapy	1.669	0.0510	5.308	0.993 - 28.320

OR, odds ratio; CI, confidence interval; The reference category is Profile 1; Profile 1: Cognitively stable but growth-constricted group; Profile 2: Post-traumatic growth but cognitively vulnerable group. Model selected by backward stepwise regression (AIC = 92.8).

The final model exhibited strong discriminatory power, with an overall accuracy of 97.2%, sensitivity of 94.0%, specificity of 98.4%, and an area under the ROC curve (AUC) of 0.992. The model also showed strong explanatory power (McFadden pseudo-R² = 0.837) and superior fit (AIC = 92.8; likelihood ratio test χ² = 242.8, P < 0.0001). Variables including household income, medical insurance type, and time since diagnosis were excluded during the stepwise selection for lacking independent predictive value. All variance inflation factors were below 2, indicating no multicollinearity concerns.

## 4 Discussion

Unlike previous variable-centered studies, this research adopted a person-centered approach to identify latent profiles of PTGI and FACT-Cog among cancer patients. The validity of this methodology in capturing meaningful psychological heterogeneity in oncology populations is well established, as exemplified by Morgan et al. (31), who identified three distinct personality profiles in patients undergoing chemotherapy. All model fit indices confirmed excellent performance (AUC = 0.992, accuracy = 97.2%; entropy > 0.8), validating the reliable two-profile solution: “cognitively stable but growth-constricted” (Profile 1) and “post-traumatic growth but cognitively vulnerable” (Profile 2). Binary logistic regression further identified key predictors for membership in Profile 2: all dimensions of the Big Five personality traits (with neuroticism as a negative predictor (OR = 0.674, 95% CI: 0.570–0.796, P < 0.001) and extraversion (OR = 1.564, 95% CI: 1.295–1.888, P < 0.001), conscientiousness (OR = 1.540, 95% CI: 1.236–1.917, P = 0.0001), agreeableness (OR = 1.346, 95% CI: 1.126–1.610, P = 0.0011), and openness as positive predictors (OR = 1.684, 95% CI: 1.289–2.209, P = 0.0001)), radiotherapy as a significant positive predictor (OR = 5.264, 95% CI: 1.033–26.850, P = 0.0458), and high educational level (OR = 5.963, 95% CI: 0.985–36.210, P = 0.0521) and endocrine therapy (OR = 5.308, 95% CI: 0.993–28.320, P = 0.0510) as marginally significant positive predictors. These findings address a critical gap by uncovering heterogeneous patterns of PTG and cognitive function, providing targeted insights for clinical intervention.

Personality traits serve as stable dispositional factors that shape coping responses to cancer-related stress, thereby exerting a profound influence on the co-development of PTGI and FACT-Cog. This perspective is strongly supported by the person-centered framework of Morgan et al. (31). In their study of 1,248 chemotherapy patients, they used LPA to identify three distinct personality profiles (“distressed,” “resilient,” and “normative”) based on the big five dimensions, demonstrating that personality-based subgrouping explains heterogeneous cancer-related outcomes. This key finding—that holistic trait configurations are paramount—directly validates our focus on personality as a core predictor of PTGI-FACT-Cog profile membership. This perspective is further supported by Liu et al. (32), who found that prostate cancer patients with cognitive impairment exhibited clustered symptom profiles closely linked to psychological vulnerability—underscoring that cognitive function in cancer populations is inherently tied to stable psychological attributes.

The significant negative association between neuroticism and Profile 2 membership (OR = 0.674, 95% CI: 0.570–0.796, P < 0.001) establishes neuroticism as a key predictor of Profile 1. This relationship can be understood through mechanisms that impede the psychological processes essential for PTG. Chapman et al. (33) demonstrated that high neuroticism correlates with negative health behaviors—including poor treatment adherence and reduced physical activity—as well as greater functional impairment in cancer patients. These factors can disrupt the cognitive reappraisal and meaning-making necessary for PTG. Furthermore, Wang et al. (34) identified a dyadic mechanism whereby patients’ neuroticism reduced both their own and their caregivers’ acceptance of illness, thereby limiting the constructive emotional processing that facilitates PTG. Interestingly, although neuroticism is typically correlated with subjective cognitive complaints, individuals in Profile 1 reported relative cognitive stability. This apparent paradox may be explained by protective factors that buffer neuroticism’s detrimental effects. Chapman et al. (33) noted that conscientiousness can mitigate the negative impact of neuroticism through structured coping and adherence to medical routines. Supporting this buffering hypothesis, Morgan et al. (31) identified a “normative” profile characterized by intermediate neuroticism coupled with moderate conscientiousness, which exhibited balanced cognitive function alongside limited PTG—a pattern strikingly similar to our Profile 1. Together, these findings suggest that neuroticism directs patients toward a growth-constricted pathway, while co-occurring protective traits may help preserve cognitive function despite limited psychological growth.

The consistent positive associations between these four traits and Profile 2 membership—extraversion (OR = 1.564, 95% CI: 1.295–1.888, P < 0.001), conscientiousness (OR = 1.540, 95% CI: 1.236–1.917, P = 0.0001), agreeableness (OR = 1.346, 95% CI: 1.126–1.610, P = 0.0011), and openness to experience (OR = 1.684, 95% CI: 1.289–2.209, P = 0.0001)—reflect their collective role in facilitating PTG while simultaneously illustrating their limited protective effect against cognitive vulnerability. This pattern can be understood through their distinct influences on psychological adaptation processes. These traits primarily operate through PTG-facilitating pathways that promote cognitive reappraisal and social engagement. Morgan et al. (31) identified a “resilient” profile characterized by high extraversion, agreeableness, and conscientiousness, which was associated with low emotional distress—creating the psychological space necessary for the meaning-making processes central to PTG. Chapman et al. (33) specifically linked conscientiousness to health-promoting behaviors that create conditions supportive of growth, while extraversion directly enhances interpersonal connectedness. The dyadic mechanisms identified by Wang et al. (34) further demonstrate how caregivers’ extraversion and conscientiousness enhance illness acceptance, thereby creating a supportive environment conducive to patients’ psychological growth. Despite their strong promotion of PTG, these personality traits have a limited capacity to prevent cognitive vulnerability. The cognitive fragility characteristic of Profile 2 primarily stems from the treatment-linked nature of Cancer-Related Cognitive Impairment (CRCI), which cannot be fully mitigated by psychological dispositions. Longitudinal evidence confirms that cognitive decline in patients is persistent and treatment-related, objectively impairing memory and executive function (35). Further mechanistic insight is provided by Ibrar et al. (36), who confirmed that CRCI affects core cognitive domains and persists in a substantial portion of patients post-treatment, attributing its causes to chemotherapy neurotoxicity and inflammatory responses—factors operating independently of personality.

Beyond personality traits, cancer treatments predicted membership in the “post-traumatic growth but cognitively vulnerable” profile. Radiotherapy significantly increased the likelihood of profile membership (OR = 5.264, 95% CI: 1.033–26.850, P = 0.0458), and endocrine therapy showed a strong positive trend (OR = 5.308, 95% CI: 0.993–28.320, P = 0.0510). These findings highlight the dual role of cancer treatments as both catalysts for psychological growth and potential contributors to cognitive vulnerability, which is supported by both established frameworks and a growing body of evidence on the heterogeneous psychological and cognitive outcomes of oncological interventions (37, 38).

The significant positive association between radiotherapy and Profile 2 membership reflects its role as a potent stressor that can foster PTG through meaning-making processes. As a newly published umbrella review confirms, radiotherapy remains a key contributor to treatment-related cognitive impairment, with its neurotoxicity disrupting neural signaling and hippocampal function (37). A patient-level meta-analysis further demonstrated that radiation for brain metastases induces initial cognitive decline, with long-term recovery rates varying by individual vulnerability (39). Moreover, long-term follow-up studies have shown that a substantial proportion of cancer survivors treated with radiotherapy face persistent cognitive challenges (40). Clinically, this dual effect necessitates integrated supportive care: for patients undergoing radiotherapy, interventions should simultaneously reinforce meaning-making (e.g., trauma-informed counseling) and address cognitive challenges (e.g., nurse-led memory training) (37, 39).

The marginal statistical significance of endocrine therapy (P = 0.0510) may stem from contextual factors such as treatment duration, type of endocrine agent, or concurrent comorbidities (41, 42). A study of long-term breast cancer survivors found that the structured self-care routines and enhanced illness awareness fostered by endocrine therapy can create a foundation for psychological growth (42). While a 6-year longitudinal study did not identify severe cognitive impairment from endocrine therapy, emerging evidence suggests subtle effects on attention and processing speed—particularly in long-term survivors (43). A nationwide cohort study further confirmed that adjuvant endocrine therapy is associated with persistent subjective cognitive complaints, which may compound with other treatment-related cognitive changes in Profile 2 patients (41). For clinical practice, this insight supports proactive monitoring of cognitive function in patients on long-term endocrine therapy, alongside tailored interventions (e.g., structured self-management programs) to capitalize on their potential for PTG while mitigating cognitive fatigue (42, 43).

The marginally significant positive predictive effect of a high educational level on Profile 2 membership (OR = 5.963, 95% CI: 0.985–36.210, P = 0.0521) suggests its potential role as an enabler of PTG. This finding aligns with cognitive reserve theory (44, 45), wherein education fosters complex cognitive frameworks that enhance capacities for cognitive reappraisal and meaning-making—core processes for deriving growth from trauma (46, 47). Clinically, individuals with high education may naturally leverage these cognitive resources in therapeutic contexts; thus, supportive interventions can focus on channeling these strengths through guided meaning-making activities (e.g., expressive writing or peer-led discussion groups). The marginal statistical significance (P = 0.0521) may reflect contextual influences such as limited sample size in the target profile (n = 67) or residual confounding from unmeasured factors (e.g., socioeconomic resources closely tied to education) (48, 49). Nevertheless, the consistent positive direction of the association reinforces the theoretical link between cognitive reserve and PTG potential. For clinical practice, this insight highlights the value of tailored support: while high-education patients may benefit from strengths-based interventions, those with lower educational attainment may require structured, accessible psychoeducational materials and nurse-led coping skills training. These targeted strategies help compensate for reduced cognitive reserve, promoting equitable psychological adaptation across educational groups.

### 4.1 Limitations and future research

There are several limitations to this study. First, the cross-sectional design precludes causal inference and cannot capture the temporal dynamics of PTG and cognitive function. Second, although key predictors were identified, the analysis did not include other potentially relevant variables—such as time since treatment completion or current treatment status—which may have influenced the findings (50). Third, the use of a convenience sample from a single region may limit generalizability. Fourth, utilizing the FACT-Cog as a self-reported outcome measure introduced inherent recall bias, unavoidably impacting the integrity of the results. A well-documented discrepancy exists between subjective and objective cognitive assessments in cancer survivors, with subjective complaints often exceeding objective impairment (51). This dissociation is particularly relevant to our findings, as factors such as depressive symptoms, anxiety, and fatigue—prevalent in this population—have been associated with over-reporting of cognitive difficulties (51). Consequently, the cognitive profiles identified may reflect not only actual cognitive function but also psychological distress and self-perceptual biases. Future studies should adopt longitudinal, multi-center designs, incorporate objective neuropsychological assessments, collect detailed treatment timeline data, and include mood measures to better elucidate the complex interplay between subjective experience and objective cognitive function in cancer survivors.

## 5 Conclusions

In summary, this study employed latent profile analysis to identify two distinct psychological adaptation patterns among cancer patients: the “cognitively stable but growth-constricted” profile and the “post-traumatic growth but cognitively vulnerable” profile. The findings demonstrate that personality traits, treatment modalities, and educational level collectively predict profile membership. Specifically, neuroticism served as a significant negative predictor, while extraversion, conscientiousness, agreeableness, and openness formed a cluster of positive predictors. Beyond personality factors, radiotherapy significantly increased the likelihood of the “post-traumatic growth but cognitively vulnerable” profile, with endocrine therapy and higher educational level showing consistent positive trends. These insights support the use of personality assessments, educational background, and treatment plans as clinical screening tools. Patients with high neuroticism or limited education represent key targets for resilience-building interventions. Those undergoing radiotherapy or possessing adaptive personality traits may benefit from integrated approaches combining strength-based psychological support with cognitive protection strategies. Additionally, patients receiving long-term endocrine therapy require proactive cognitive monitoring. Collectively, these tailored strategies aim to enhance psychological resilience and improve overall quality of life.

## Data Availability

The original contributions presented in the study are included in the article/supplementary material. Further inquiries can be directed to the corresponding author/s.
